# Clinical Effectiveness of Resin Infiltration and Fluoride Varnish for Managing White Spot Lesions (International Caries Detection and Assessment System (ICDAS) ≤ 2) During Multibracket Orthodontic Treatment: A Systematic Review and Meta-Analysis

**DOI:** 10.7759/cureus.107633

**Published:** 2026-04-24

**Authors:** Vilius Kosys, Gabija Streimikyte, Giedre Trakiniene

**Affiliations:** 1 Odontology, Lithuanian University of Health Sciences, Kaunas, LTU; 2 Orthodontics, Lithuanian University of Health Sciences, Kaunas, LTU

**Keywords:** fluoride varnish, icdas, orthodontics brackets, resin infiltration, white spot lesions (wsls)

## Abstract

White spot lesions (WSLs) are a common adverse effect of fixed orthodontic treatment, caused by enamel demineralization associated with biofilm accumulation around brackets. These lesions are typically non-cavitated. Conventional treatments such as fluoride varnish enhance remineralization but often provide limited esthetic improvement. Resin infiltration has emerged as a minimally invasive alternative that improves the appearance of lesions by infiltrating porous enamel and altering its optical properties. This systematic review and meta-analysis evaluated the clinical effectiveness of resin infiltration compared with fluoride varnish in the management of WSLs (International Caries Detection and Assessment System (ICDAS) ≤ 2) in orthodontic patients.

A systematic search was conducted across five electronic databases from November 2020 to December 2025. Randomized controlled trials comparing resin infiltration with fluoride varnish or placebo were included. The primary outcome was esthetic improvement assessed by color change (ΔE). Secondary outcomes included lesion regression, changes in ICDAS scores, and caries activity. A random-effects meta-analysis was performed using IBM SPSS 30.0 (IBM Corp., Armonk, NY, USA), and results were expressed as mean differences (MDs) with 95% confidence intervals (CIs).

Three randomized controlled trials were included, of which two were eligible for meta-analysis. Meta-analysis showed a statistically significant reduction in color difference favoring resin infiltration (MD = −3.43; 95% CI: −4.20 to −2.66; I² = 0%). Narrative synthesis indicated greater lesion regression and caries arrest in the resin infiltration group, reflected by reductions in ICDAS scores and caries activity measures, including quantitative light-induced fluorescence (QLF) and DIAGNOdent values. Follow-up periods ranged from one week to 12 months.

This review is subject to several limitations, including a restriction to English-language publications, a limited publication timeframe (2020-2025), and the inclusion of only full-text studies, which may have introduced selection bias and limited the comprehensiveness of the evidence. Furthermore, gray literature sources and trial registries were not systematically searched, which may have resulted in the omission of relevant unpublished or ongoing studies. The small number of included studies and relatively short follow-up periods may limit the generalizability of the findings.

Resin infiltration appears to be more effective than fluoride varnish in improving esthetic outcomes and promoting regression of ICDAS ≤ 2 WSLs. It represents a valuable minimally invasive treatment option; however, further well-designed randomized controlled trials with longer follow-up are required to confirm long-term effectiveness.

## Introduction and background

Enamel opacities may develop as a result of injury to the dental follicle during eruption, irregularities in enamel maturation, or the effects of cariogenic processes associated with poor oral hygiene (e.g., increased plaque accumulation) [1]. Fixed orthodontic appliances are associated with this phenomenon because they create additional retention sites for biofilm accumulation, thereby increasing the risk of caries [2,3]. These lesions, characterized by a whitish, opaque, and chalky appearance due to enamel demineralization [4], are referred to as white spot lesions (WSLs).

The detection of non-cavitated caries in clinical practice remains challenging. While visual inspection is widely used as the primary diagnostic approach, adjunctive methods such as quantitative light-induced fluorescence (QLF), DIAGNOdent, and bitewing radiography can improve diagnostic accuracy and reduce subjectivity. Compared with the WHO criteria, the International Caries Detection and Assessment System (ICDAS) is preferred for evaluating both early non-cavitated and cavitated lesions, as it allows more precise detection of initial caries [5,6].

Fluoride-based products are among the most widely used preventive agents for managing WSLs and early carious lesions [6,7]. Their primary mechanism involves enhancing enamel remineralization and inhibiting demineralization, thereby increasing resistance to acid challenges from cariogenic bacteria [7]. Other approaches, such as resin infiltration (RI), can also improve the esthetic appearance of WSLs [8]. RI involves filling the microporous enamel regions of non-cavitated, initial carious lesions with low-viscosity, light-cured resins (infiltrants) [9]. The refractive index of the infiltrant (1.52) is close to that of enamel/apatite (1.62), unlike those of water (1.33) and air (1.00), resulting in reduced light scattering and improved visual appearance [10,11].

Randomized studies suggest that RI may mask WSLs more effectively than fluoride varnish (FV) [12]. Previous systematic reviews, including Bourouni et al., have evaluated RI across broader clinical contexts and a larger number of studies, generally reporting favorable outcomes compared with remineralization-based approaches [13]. However, these reviews included heterogeneous populations, lesion severities, and study designs, which may limit their direct applicability to early non-cavitated lesions in orthodontic patients.

Therefore, the aim of this systematic review and meta-analysis was to evaluate the clinical effectiveness of RI compared with FV, specifically in the management of ICDAS ≤ 2 WSLs in patients undergoing fixed orthodontic treatment. By focusing on early-stage lesions, a homogeneous orthodontic population, and recent clinical evidence, this review seeks to provide a more targeted and clinically relevant synthesis of the available data.

The study protocol was registered at the International Prospective Registry of Systematic Reviews (PROSPERO) database (registration number: CRD420251239505).

## Review

Materials and methods

Systematic Review Protocol and Registration

This systematic review followed the Preferred Reporting Items for Systematic Reviews and Meta-analyses (PRISMA) guidelines [14]. The aim of the review was to evaluate the clinical effectiveness of RI and FV in managing ICDAS ≤ 2 WSLs in patients undergoing multibracket orthodontic treatment. The study protocol was registered at the International Prospective Registry of Systematic Reviews (PROSPERO) database (registration number: CRD420251239505). The review protocol was developed using the PICO (Population, Intervention, Comparison, and Outcome) framework. The full question formulation is presented in Table 1.

**Table 1:** Description of the research question formulated using the PICO framework ICDAS: International Caries Detection and Assessment System

PICO element	Description
P (Population)	Human patients of any sex and ethnicity up to 70 years old undergoing fixed orthodontic appliances (brackets), who have or are at risk of developing enamel white spot lesions (WSLs) classified as ICDAS ≤ 2
I (Intervention)	Resin infiltration and fluoride varnish as interventions for the management of ICDAS ≤ 2 WSLs in patients undergoing fixed multibracket orthodontic treatment
C (Comparison)	Resin infiltration versus fluoride varnish
O (Outcomes)	Change in WSL severity during multibracket orthodontic treatment
Research question	What is the clinical effectiveness of resin infiltration between fluoride varnish in managing ICDAS ≤ 2 WSLs in patients undergoing multibracket orthodontic treatment?

Search Strategy

The literature search was conducted in five electronic databases: PubMed, Web of Science, ScienceDirect, SpringerLink, and Wiley Online Library. The search covered the period from November 9, 2020, to December 1, 2025, with the final search performed on December 10, 2025. This timeframe was selected to capture the most recent evidence reflecting contemporary clinical practices and materials. Only studies published in English were included due to feasibility constraints and to ensure accurate data interpretation. Boolean operators (AND, OR) and Medical Subject Headings (MeSH) were used to refine the search.

The search string was as follows: (("white spot lesion" OR "white spot lesions" OR "demineralization") AND (ICDAS OR "International Caries Detection and Assessment System") AND ("resin infiltration") AND ("fluoride varnish") AND (orthodontics* OR "fixed appliance" OR multibracket)). Searches were performed using a combination of controlled vocabulary (MeSH terms) and free-text terms. Field tags (e.g., title/abstract) were applied where appropriate, and truncation symbols were used to capture variations of key terms. For example, the PubMed search strategy was as follows: (("white spot lesion"[Title/Abstract] OR "white spot lesions"[Title/Abstract] OR demineralization[Title/Abstract]) AND (ICDAS OR "International Caries Detection and Assessment System") AND ("resin infiltration"[Title/Abstract]) AND ("fluoride varnish"[Title/Abstract]) AND (orthodontics[Title/Abstract] OR "fixed appliance"[Title/Abstract] OR multibracket[Title/Abstract])). No additional filters were applied other than language (English) and publication date (2020-2025).

After initial screening of titles and abstracts, eligible full-text articles were assessed for inclusion. Duplicates were removed using Mendeley software (Elsevier, Amsterdam, Netherlands). Gray literature sources and trial registries were not systematically searched.

Eligibility Criteria and Study Selection

Studies were included if they (1) were clinical studies (randomized controlled trials (RCTs) and controlled clinical trials (CCTs)) conducted on human patients of any sex and ethnicity, aged up to 70 years, undergoing fixed orthodontic treatment (brackets) who had or were at risk of developing enamel WSLs classified as ICDAS I or II and received RI and FV; (2) were published in English between 2020 and 2025; and (3) had full-text availability. Non-randomized studies, in vitro/in situ/animal studies, case reports, case series, and systematic literature reviews were excluded. Participants not undergoing fixed orthodontic treatment and non-human subjects were excluded. Studies evaluating only other interventions (e.g., mechanical plaque control, antibacterial mouth rinses, and lasers) without RI or FV were excluded. Additionally, studies not reporting visual or mineralization outcomes of WSL, studies without ICDAS assessment, or studies including lesions with ICDAS scores greater than 2 were not considered.

Data Extraction

Data extraction was performed independently by two reviewers. Any disagreements were resolved through discussion and, when necessary, by consultation with a third reviewer. From each included study, the following information was systematically extracted: the authors and year of publication, the study design (e.g., RCT and CCT), and the sample size. Details regarding the diagnostic criteria for WSLs were recorded, including whether lesions were identified using ICDAS scores ≤ 2, visual-tactile examination, or other clinical assessment methods. Information on the intervention type was collected, specifying whether RI or FV was applied, including details on frequency, application protocol, and duration of follow-up. Finally, the outcomes of interest were extracted, such as lesion progression or regression, esthetic improvement (e.g., ΔE color change), and any reported adverse effects.

Bias Assessment

Study quality was assessed using the Joanna Briggs Institute (JBI) Critical Appraisal Checklist for randomized controlled studies [15]. “Yes,” “no,” or “unclear” was assigned to each criterion. Methodological quality was assessed as follows: high risk of bias for studies with 49% or fewer positive responses, moderate risk of bias for 50%-69%, and low risk of bias for more than 70%. Assessment of publication bias (e.g., funnel plot analysis) was not performed due to the small number of included studies.

All included RCTs achieved more than 70% positive ratings across risk-of-bias assessment items, indicating an overall low risk of bias. The quality of the included studies is summarized in Table 2.

**Table 2:** Results of randomized controlled studies from the Joanna Briggs Institute Critical Appraisal Checklist Appraisal checklist: (+) yes; (−) no RCT: randomized controlled trial

Study	Year	Study design	Checklist												
			Q1	Q2	Q3	Q4	Q5	Q6	Q7	Q8	Q9	Q10	Q11	Q12	Q13
Wierichs et al. [16]	2023	RCT	+	+	+	-	-	+	+	+	+	+	+	+	+
Wierichs et al. [17]	2023	RCT	+	+	+	-	-	+	+	+	+	+	+	+	+
Kashash et al. [18]	2024	RCT	+	+	+	-	-	+	+	+	+	+	+	+	+

Risk-of-Bias Results

The methodological quality of the included RCTs was evaluated using the JBI Critical Appraisal Checklist for RCTs. Although all three studies scored above 70% based on predefined thresholds, a domain-based assessment revealed some potential sources of bias. In particular, performance bias could not be excluded, as blinding of participants and clinicians was not feasible due to the nature of the interventions (e.g., RI versus FV). Outcome assessor blinding was reported in all studies, which may have reduced detection bias. Other domains, including randomization, allocation concealment, standardized outcome assessment, and completeness of follow-up, were generally well addressed. Overall, while the included studies demonstrated moderate to high methodological quality, the risk of bias should be interpreted with caution, particularly due to the lack of blinding and the limited number of included studies.

Data synthesis

Results

**Figure 1:**
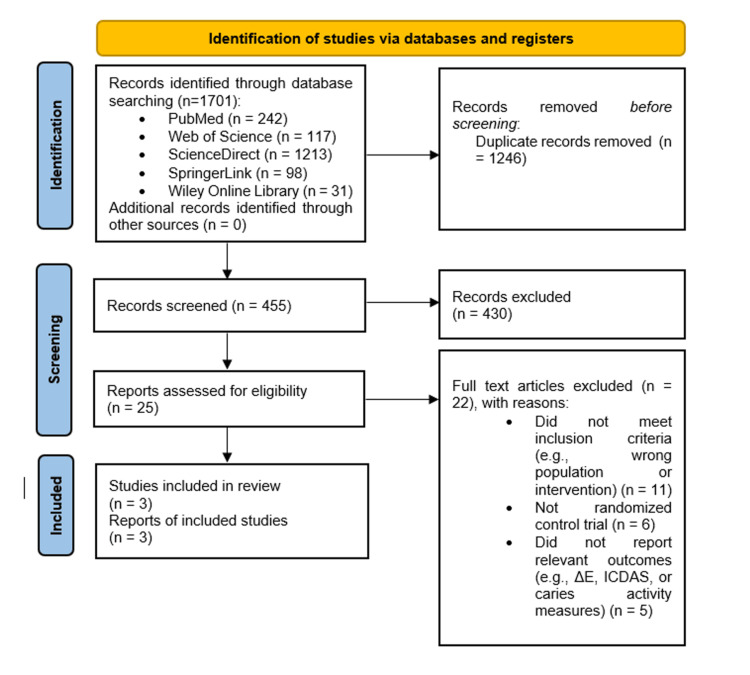
PRISMA flow chart PRISMA: Preferred Reporting Items for Systematic Reviews and Meta-analyses; ICDAS: International Caries Detection and Assessment System

Study selection: A total of 1,701 records were identified through database searching. After removing 1,246 duplicate records, 455 records remained for title and abstract screening. Of these, 430 were excluded as irrelevant. Twenty-five full-text articles were assessed for eligibility, and 22 were excluded for the following reasons: did not meet inclusion criteria (e.g., wrong population or intervention) (n = 11), not RCT (n = 6), did not report relevant outcomes (e.g., ΔE, ICDAS, or caries activity measures) (n = 5). Finally, three RCTs [16-18] met the inclusion criteria and were included in the qualitative synthesis, of which two were eligible for meta-analysis due to the availability of comparable outcome data. The study selection process is summarized in the PRISMA flow diagram (Figure 1).

The main reasons for exclusion at the full-text stage included non-randomized study design, lack of relevant outcome measures (such as ΔE, ICDAS scores, or caries activity indices), and failure to meet predefined inclusion criteria related to population, intervention, or study design. In total, three RCTs were included in this systematic review and meta-analysis. The main characteristics of the included studies are summarized in Table 3. All studies assessed the clinical effectiveness of RI compared with FV for the management of ICDAS ≤ 2 WSLs during multibracket orthodontic treatment.

**Table 3:** Characteristics of the included studies ICDAS: International Caries Detection and Assessment System

Study	Sample	Intervention	Comparator	Follow-up	Outcomes
Wierichs et al. [16]	15 patients	Resin infiltration	Fluoride varnish	1 week	ΔE, ICDAS, DIAGNOdent
Wierichs et al. [17]	15 patients	Resin infiltration	Fluoride varnish	Until debonding (~6 months)	ΔE, ICDAS, DIAGNOdent
Kashash et al. [18]	36 patients	Resin infiltration	Fluoride varnish	6 months	ΔE, ICDAS

Two studies used a split-mouth design [16,17], while one employed a parallel-group design [18]. The duration of follow-up ranged from one week to approximately 12 months or until appliance debonding.

Quantitative meta-analysis

Esthetic Outcome (ΔE)

Two RCTs [17,18] were eligible for quantitative synthesis, as they reported comparable ΔE outcomes at similar follow-up periods. A meta-analysis was performed using a random-effects model to account for potential clinical and methodological heterogeneity among the included studies, despite low statistical heterogeneity (I² = 0%). However, the results should be interpreted with caution, given the small number of included studies. Statistical analyses were performed using IBM SPSS Statistics for Windows, version 30.0 (IBM Corp., Armonk, NY, USA). RI demonstrated a statistically significant reduction in ΔE values compared with FV: mean difference (MD): −3.43; 95% confidence interval (CI): −4.20 to −2.66; statistical heterogeneity (I²): 0%.

Forest Plot

**Figure 2:**
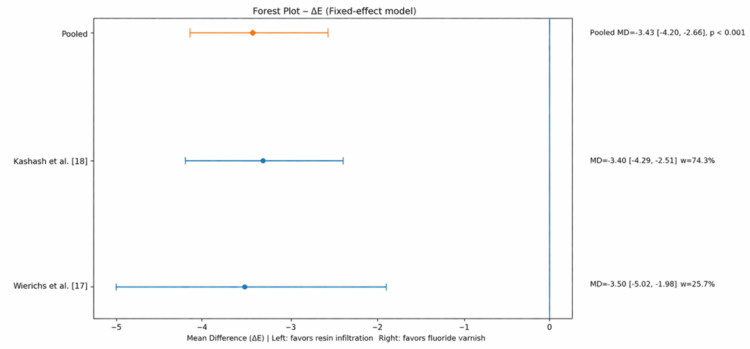
Forest plot comparing resin infiltration and fluoride varnish for esthetic outcome (ΔE). A random-effects model was used. Both studies demonstrated lower ΔE values favoring resin infiltration. The pooled mean difference (MD) was −3.43 (95% CI −4.20 to −2.66; p < 0.001). No heterogeneity was observed (I² = 0.0%, τ² = 0.00, Q = 0.01) CI: confidence interval

The results of the meta-analysis are presented in Figure 2.

The forest plot revealed a consistent treatment effect in favor of RI across both included studies. All effect estimates were located on the left side of the line of no effect (MD = 0), indicating lower ΔE values and therefore superior esthetic outcomes in the RI group.

The CIs did not cross the null line, confirming statistical significance. Additionally, no heterogeneity was observed (I² = 0.0%, τ² = 0.00, Q = 0.01), suggesting a high level of consistency between studies.

Narrative synthesis

Esthetic Improvement

All included studies [16-18] reported a trend toward greater esthetic improvement in lesions treated with RI compared with FV. However, the magnitude and timing of this effect varied across studies.

Split-mouth and randomized designs demonstrated a reduction in ΔE values following RI, in some cases, observable shortly after treatment. In contrast, FV was generally associated with more limited or delayed esthetic improvement. At medium-term follow-up (approximately six months or until debonding), RI appeared to maintain a relatively stable esthetic effect, although differences in study design, follow-up duration, and outcome assessment methods limit direct comparability.

ICDAS Regression and Caries Arrestment

All studies assessing ICDAS outcomes [16-18] indicated a tendency toward greater lesion regression and caries arrest following RI compared with FV. Lesions treated with RI showed a shift toward lower ICDAS scores (ICDAS 1 or 0), suggesting lesion inactivation. However, variability in diagnostic criteria and follow-up duration may have influenced the reported degree of regression. In addition, the lack of participant and operator blinding may have introduced performance bias, particularly in visually assessed outcomes such as ICDAS scoring.

Additional Measures of Caries Activity

Studies reporting DIAGNOdent and QLF outcomes [16-18] generally demonstrated reductions in caries activity following RI compared with FV. However, these findings should be interpreted with caution due to differences in measurement techniques, small sample sizes, and the limited number of studies reporting these outcomes.

Overall Results

The combined evidence from quantitative meta-analysis and narrative synthesis indicates that RI is superior to FV for the management of ICDAS ≤ 2 WSLs during multibracket orthodontic treatment. RI provides greater and faster esthetic improvement, enhanced ICDAS regression, and more effective caries arrestment throughout the course of orthodontic therapy. A summary of esthetic and clinical outcomes across the included studies is presented in Table 4.

**Table 4:** Summary of esthetic and clinical outcomes [16-18] ICDAS: International Caries Detection and Assessment System; QLF: quantitative light-induced fluorescence

Outcome	Evidence base	Main finding
ΔE (esthetic improvement)	Meta-analysis and narrative synthesis	Significant and sustained reduction favoring resin infiltration
ICDAS regression	Narrative synthesis	Greater regression with resin infiltration
Caries arrestment	Narrative synthesis	Earlier and more pronounced arrestment with resin infiltration
DIAGNOdent/QLF	Narrative synthesis	Greater reduction in caries activity with resin infiltration

Discussion

This systematic review suggests that RI may be more effective than FV in improving the visual appearance and clinical behavior of ICDAS ≤ 2 WSLs during fixed orthodontic treatment. Although only two RCTs provided suitable data for quantitative synthesis, this study showed a statistically significant reduction in ΔE favoring RI at six months (MD = −3.43; 95% CI: −4.20 to −2.66), with no observed heterogeneity (I² = 0%). This finding suggests a potential esthetic advantage, although it should be interpreted with caution given the limited number of studies.

It should be noted that two included studies used a split-mouth design, which may introduce within-subject correlation and affect the independence of observations. Due to limited reporting of paired data, adjustments for clustering were not performed, and the results should therefore be interpreted with caution.

The narrative synthesis further supports this conclusion. Across all included studies [16-18], RI resulted in significantly greater esthetic improvement compared with FV, with a pooled MD (ΔE) of −3.43 (95% CI: −4.20 to −2.66; p < 0.001), with visible lesion masking sometimes observed as early as one week in some included studies, although follow-up periods varied. In contrast, FV resulted in minimal or delayed esthetic improvement. These differences can be explained by the underlying mechanisms of action. Resin infiltrants penetrate the porous lesion body and replace air-water interfaces with a resin matrix whose refractive index closely approximates that of sound enamel [9,10,13]. This reduces internal light scattering and immediately enhances optical blending with adjacent enamel, producing effective lesion masking. FV primarily acts by promoting surface remineralization and lesion stabilization rather than modifying the optical properties of the subsurface lesion, which limits its ability to restore enamel translucency [2,3].

Clinical outcomes beyond esthetics also favored RI. Greater lesion regression and caries arrest were observed in the RI group, as reflected by reductions in ICDAS scores and caries activity measures, including QLF and DIAGNOdent values [16-18]. Lesions treated with RI demonstrated a consistent shift toward lower ICDAS scores (ICDAS 1 or 0), indicating lesion inactivation, whereas FV may be associated with a trend toward slower and less pronounced improvement. Additional diagnostic measures, such as DIAGNOdent and QLF [16-18], supported these results by showing greater reductions in lesion activity following RI. These findings suggest that RI may provide both optical and caries-arresting benefits.

The results of this review are consistent with previous research demonstrating superior masking and clinical performance of RI compared with remineralization-based approaches [13,19-23]. However, FV remains an important preventive therapy, particularly in orthodontic patients who are at increased risk of enamel demineralization due to biofilm accumulation [2,4,7,17]. Therefore, RI should be considered a minimally invasive treatment option for established lesions rather than a substitute for caries-preventive measures.

The certainty of the evidence was assessed using the GRADE (Grading of Recommendations Assessment, Development, and Evaluation) approach. Due to the small number of included studies, limited sample sizes, and potential risk of bias (particularly performance bias), the overall certainty of the evidence was rated as low to moderate. The evidence for esthetic outcomes (ΔE) was considered of moderate certainty, while evidence for secondary outcomes (ICDAS, QLF, and DIAGNOdent) was considered low certainty due to heterogeneity in outcome assessment and limited reporting.

Despite the strength of consistent findings across studies, some limitations should be acknowledged. Two RCTs contributed data to the quantitative meta-analysis, which limits the strength of the pooled statistical inference. Follow-up periods varied across studies, ranging from one week to 12 months, which may have introduced heterogeneity and affected the comparability of results; although short- and medium-term outcomes favored RI, long-term color stability and lesion behavior remain uncertain and require further investigation. Blinding of operators and patients was generally not feasible due to the obvious procedural differences between interventions, which may introduce performance bias. Nevertheless, outcome assessor blinding was applied where possible; however, conclusions regarding overall methodological quality should be interpreted with caution, given the small number of included studies and the limited evidence base.

WSLs remain a frequent clinical challenge during orthodontic treatment, with high reported prevalence in patients with fixed appliances [2-4]. Early diagnosis using structured systems such as ICDAS supports targeted clinical decision-making [1,5]. Within this preventive framework, RI represents a valuable micro-invasive treatment approach that addresses both the biological and esthetic components of early enamel lesions.

From a clinical perspective, RI offers immediate esthetic improvement and lesion stabilization; however, it may be associated with higher costs and longer chairside time compared with FV application. In addition, the technique is more operator-sensitive and requires strict moisture control, which may influence its applicability in routine clinical settings. Patient acceptance is generally high due to the minimally invasive nature of the procedure and the immediate improvement in appearance, although accessibility and cost may limit widespread use.

In summary, the findings of this systematic review indicate that RI provides superior esthetic improvement and greater lesion regression compared with FV in managing ICDAS ≤ 2 WSLs during fixed orthodontic treatment. Future research should prioritize well-designed RCTs with longer follow-up periods, standardized lesion assessment protocols, and evaluation of patient-reported outcomes to further clarify the long-term clinical benefits of RI. Overall, while the findings consistently favor RI, the limited number of studies and methodological variability reduce the certainty of the evidence and highlight the need for cautious interpretation.

## Conclusions

Within the limitations of the included studies, this systematic review indicates that RI is more effective than FV in improving the visual appearance of ICDAS 1-2 WSLs during fixed orthodontic treatment. Quantitative meta-analysis based on comparable ΔE outcome data demonstrated a statistically significant greater reduction in color difference in favor of RI (MD = −3.43; 95% CI: −4.20 to −2.66), exceeding commonly accepted thresholds for clinical perceptibility and indicating superior masking of lesions. Narrative findings further supported these results, showing greater ICDAS regression, earlier lesion arrest, and larger reductions in caries activity measures (such as DIAGNOdent and QLF values) following RI compared with FV.

While FV remains an important preventive and remineralization therapy for lesion control, its esthetic effect is limited when compared with RI. RI may offer a micro-invasive treatment option for established WSLs with esthetic concerns in orthodontic patients; however, further high-quality studies are required to confirm its effectiveness. Further well-designed RCTs with standardized outcome measures, longer follow-up periods, and patient-reported outcome assessment are required to confirm the long-term durability and clinical impact of these findings.

## References

[1] Ekstrand KR, Martignon S (2013). Visual-tactile detection and assessment. Caries Management-Science and Clinical Practice.

[2] Hadler-Olsen S, Sandvik K, El-Agroudi MA, Øgaard B (2012). The incidence of caries and white spot lesions in orthodontically treated adolescents with a comprehensive caries prophylactic regimen--a prospective study. Eur J Orthod.

[3] Gorelick L, Geiger AM, Gwinnett AJ (1982). Incidence of white spot formation after bonding and banding. Am J Orthod.

[4] Heymann GC, Grauer D (2013). A contemporary review of white spot lesions in orthodontics. J Esthet Restor Dent.

[5] Frencken JE, Giacaman RA, Leal SC (2020). An assessment of three contemporary dental caries epidemiological instruments: a critical review. Br Dent J.

[6] Butera A, Maiorani C, Morandini A, Simonini M, Morittu S, Trombini J, Scribante A (2022). Evaluation of children caries risk factors: a narrative review of nutritional aspects, oral hygiene habits, and bacterial alterations. Children (Basel).

[7] Derks A, Katsaros C, Frencken JE, van't Hof MA, Kuijpers-Jagtman AM (2004). Caries-inhibiting effect of preventive measures during orthodontic treatment with fixed appliances. A systematic review. Caries Res.

[8] Kobbe C, Fritz U, Wierichs RJ, Meyer-Lueckel H (2019). Evaluation of the value of re-wetting prior to resin infiltration of post-orthodontic caries lesions. J Dent.

[9] Meyer-Lueckel H, Paris S (2008). Improved resin infiltration of natural caries lesions. J Dent Res.

[10] Wierichs RJ, Kogel J, Lausch J, Esteves-Oliveira M, Meyer-Lueckel H (2017). Effects of self-assembling peptide P11-4, fluorides, and caries infiltration on artificial enamel caries lesions in vitro. Caries Res.

[11] Houwink B (1974). The index of refraction of dental enamel apatite. Br Dent J.

[12] Wang Q, Zhou Y, Cui T, Li J, Lo EC, Hao G, Zhi Q (2023). Comparative evaluation of four treatments for postorthodontic white spot lesions: a randomized controlled trial. Clin Oral Investig.

[13] Bourouni S, Dritsas K, Kloukos D, Wierichs RJ (2021). Efficacy of resin infiltration to mask post-orthodontic or non-post-orthodontic white spot lesions or fluorosis-a systematic review and meta-analysis. Clin Oral Investig.

[14] Page MJ, McKenzie JE, Bossuyt PM (2021). The PRISMA 2020 statement: an updated guideline for reporting systematic reviews. BMJ.

[15] Aromataris E, Lockwood C, Porritt K, Pilla B, Jordan Z (2024). JBI Manual for Evidence Synthesis. JBI Manual for Evidence Synthesis. Joanna Briggs Institute.

[16] Wierichs RJ, Bourouni S, Kalimeri E, Gkourtsogianni S, Meyer-Lueckel H, Kloukos D (2023). Short-term efficacy of caries resin infiltration during treatment with orthodontic fixed appliances. A randomized controlled trial. Eur J Orthod.

[17] Wierichs RJ, Selzner H, Bourouni S, Kalimeri E, Seremidi K, Meyer-Lückel H, Kloukos D (2023). Masking-efficacy and caries arrestment after resin infiltration or fluoridation of initial caries lesions in adolescents during orthodontic treatment-a randomised controlled trial. J Dent.

[18] Kashash Y, Hein S, Göstemeyer G, Aslanalp P, Weyland MI, Bartzela T (2024). Resin infiltration versus fluoride varnish for visual improvement of white spot lesions during multibracket treatment. A randomized-controlled clinical trial. Clin Oral Investig.

[19] Knösel M, Eckstein A, Helms HJ (2013). Durability of esthetic improvement following Icon resin infiltration of multibracket-induced white spot lesions compared with no therapy over 6 months: a single-center, split-mouth, randomized clinical trial. Am J Orthod Dentofacial Orthop.

[20] Paris S, Meyer-Lueckel H (2009). Masking of labial enamel white spot lesions by resin infiltration--a clinical report. Quintessence Int.

[21] Son JH, Hur B, Kim HC, Park JK (2011). Management of white spots: resin infiltration technique and microabrasion. Restor Dent Endod.

[22] Sonesson M, Bergstrand F, Gizani S, Twetman S (2017). Management of post-orthodontic white spot lesions: an updated systematic review. Eur J Orthod.

[23] Marinelli G, Inchingolo AD, Inchingolo AM (2021). White spot lesions in orthodontics: prevention and treatment. A descriptive review. J Biol Regul Homeost Agents.

